# Recovery of electro-mechanical properties inside self-healing composites through microencapsulation of carbon nanotubes

**DOI:** 10.1038/s41598-020-59725-6

**Published:** 2020-02-19

**Authors:** Hasna Hena Zamal, David Barba, Brahim Aïssa, Emile Haddad, Federico Rosei

**Affiliations:** 1 Institut National de la Recherche Scientifique, Centre: Energie, Matériaux et Télécommunications, 1650 Boulevard Lionel-Boulet, Varennes, Québec J3X 1S2 Canada; 2 MPB Communications Inc., Space & Photonics Division, 151 Hymus Boulevard, Pointe Claire, Montréal, QC H9R 1E9 Canada; 3 Qatar Environment and Energy Research Institute (QEERI), Hamad Bin Khalifa University, Qatar Foundation, 5825 Doha, State of Qatar

**Keywords:** Engineering, Materials science

## Abstract

We report the successful microencapsulation of multi-walled carbon nanotubes suspended in a 5-ethylidene-2-norbornene (5E2N) self-healing monomer, into poly melamine urea formaldehyde shells through *in situ* polymerization. The average size of the microcapsules, their size-distribution, shell wall structural integrity and thickness are  characterized by optical and scanning electron microscopy. The presence of carbon nanotubes (CNTs) inside the core liquid content, as well as their release after breaking is confirmed by microscopy and spectroscopy analyses. A small amount of CNTs inside the microcapsules is found to have no significant impact on the thermal stability of the system, as determined by thermogravimetric analysis and differential scanning calorimetry. Both the mechanical and the electrical properties of CNT-based self-healing materials can be restored  up to 80% when CNT/5E2N microcapsules are incorporated into polymer composites, thus making them highly suitable for applications in aerospace.

## Introduction

Key mechanical structures and electronic components of modern airplanes and spacecrafts are made up of polymer composites^1^, due to their high strength-to-weight ratio and durability^2^. Some devices, such as space antenna, require to keep intact both their mechanical and electrical properties during their operation lifetime. However, harsh environmental conditions^3^ and/or collisions with space debris can seriously compromise their structural integrity and/or electrical conductivity^4^.

Self-healing is a process by which a damaged material can partially or totally restore its original properties after degradation and/or the occurrence of accidental events^5–7^. Such a functionality is very attractive to extend the lifetime of devices and systems launched in space, such as telecommunication satellites, orbital segments and exploration modules. The concept of self-healing polymers is based on the incorporation of healing reagents stored in micro storage vessels or microcapsules, into polymeric composites. This class of advanced materials has been widely studied after the pioneering work of White *et al*.^8^, who showed the efficient recovery of mechanical properties in epoxy-based composite systems containing liquid Dicyclopentadiene (DCPD) monomers capable of undergoing ring opening metathesis polymerization (ROMP) reaction using the Ruthenium Grubbs catalyst as initiator^9^. The ROMP is a chain growth metathesis polymerization reaction of cyclic olefins (e.g. Ethylidene Norbornene) in which the reaction is driven from monomer to polymer by the release of ring strain associated with the cyclic olefin. During the ROMP process, the double bond (or any unsaturation) present in the reactant monomer participates in the reaction but it is finally conserved in the produced polymer. This enables the synthesis of polymer with unique characteristics^9^. The ROMP of a suitable monomer, such as 5E2N, can also simply be initiated quickly at room temperature (RT)  and even at sub-zero temperatures by a suitable catalyst, such as Hoveyda-Grubbs catalyst (HG2)^10^. These features of ROMP differentiate them from other typical polymerization processes (such as ethylene-polyethylene)^9^ and make it suitable for self-helaing applicaitons.

Until now, many efforts were made to microencapsulate other healing agent monomers and polymerization initiators to impart self-healing functionality to these materials for various applications^11–15^. Among them, the DCPD/Grubbs catalysts have been investigated extensively due to their huge potential^16–24^, but as the DCPD freezing point is 15 °C, this strongly limits their use in low temperature environments where aerospace vehicles are usually operated^25^. Such a constraint motivates the development of alternative healing agent monomers, leading to similar ROMP reaction with Grubbs catalyst and remaining fluid at lower temperatures. Among other promising candidates, 5-ethylidene-2-nobornone (5E2N) is attractive because this compound freezes around −80 °C^25^ and undergoes a much faster ROMP reaction with the Grubbs catalyst (around few seconds at  RT), using much less catalyst loading (as low as 0.1 wt. %) compared to DCPD^26^.

Previous works conducted on the 5E2N healing agent have shown that liquid 5E2N can be successfully microencapsulated into poly melamine urea formaldehyde (PMUF) shells using *in situ* polymerization techniques^27,28^ In addition to this, our investigations of the ROMP kinetics of 5E2N with HG2 catalyst using Raman spectroscopy have clearly demonstrated the relevance of using these compounds for self-healing applications at temperatures as low as −30 °C^10^. However, unlike DCPD, 5E2N produces a polymer that is less mechanically resistant upon ROMP reaction. To overcome this drawback, the incorporation of carbon nanotubes (CNTs) into 5E2N has been suggested^25^. The addition of 0.4 wt.–0.8 wt.% norbornene-functionalized CNTs has been found to significantly increase the tensile toughness of DCPD and 5E2N polymers prepared by ROMP^29,30^. A small amount of CNTs (0.1‒6 wt. %) in epoxy composites also enhances the mechanical strength and shear modulus of the epoxy^31–33^, as well as its electrical conductivity by several orders of magnitude^34^ and its thermal conductivity by up to 300%^35–39^.

Here, we report the successful microencapsulation of CNT/5E2N suspension and we characterize the ability of this system to restore autonomously both its mechanical and electrical properties inside damaged polymer composites. As the ultimate purpose of our work is to elaborate self-healing composite materials offering a fast ROMP reaction, with high recovery rate of electro/mechanical properties, we implemented different procedures to encapsulate the CNT/5E2N suspension in different weight ratios and investigated their healing efficiency. To demonstrate the self-healing capabilities of microencapsultated CNT/5E2N, fracture tests and electrical conductivity restoration tests are performed, showing that these compounds can be used to restore up to 80% of the fracture toughness after complete failure, and 82% of the electrical conductivity after the conductive path of self-healing polymer samples was interrupted.

## Conceptual Route of Self-Healing with CNT/5E2N Microcapsules

The successful microencapsulation of CNTs with self-healing monomer, such as 5E2N, can open multiple opportunities for self-healing applications in space environment. In addition to making polymer composites more resistant mechanically^40–45^, the use of CNTs can improve the electrical conductivity of the composites^38,40,41,46^, avoid sparking in fuel filter housings^47,48^, act as sensors for monitoring damages in high performance batteries^49–51^, as well as contribute to the development of innovative electronic devices, such as circuits printed on flexible sheets^52^, anticorrosive coatings and conductive adhesives^11^. Recently, CNTs have been increasingly tested in various components of future generation aircrafts in which polymer composites are already used to make a majority of their components and structures^2^. As some of commercial passenger airplanes (Boeing 787, Airbus A350 and Bombardier Cseries models) presently employ polymer composites for up to 50% of their total weight^2^, the microencapsulation of CNT/5E2N suspensions inside these materials could give them self-healing abilities that would improve both their reliability and durability.

Caruso *et al*.^13^ successfully synthesized microcapsules containing single wall carbon nanotubes (SWCNTs) mixed with organic solvents inside poly urea-formaldehyde (PUF) vessels to restore the electrical properties of various polymeric matrices. However, the SWCNTs used in these suspensions did not disperse efficiently within the solvents, leading to aggregation^13^. Similarly, Pastine *et al*.^53^ encapsulated 1 wt. % of multiwall carbon nanotubes (MWCNTs) suspended in toluene inside polyamide shells by interfacial polymerization, which is intended to produce remotely triggerable microcapsules. In these reports^13,50,53^, however, the majority of core materials embedded within these microcapsules were solvents, which cannot undergo polymerization reaction for self-healing of cracks or damages of composites.

Regarding the healing agent, Bailey *et al*.^54^ were able to encapsulate epoxy with high concentration (up to 20 wt. %) of CNTs using ethyl-phenyl acetate (EPA) solvent for conductive coating applications. A conductivity recovery of 64% (±23%) and a structural recovery of 81% (±39%) were measured by *in situ* electro-tensile tests conducted on coatings and substrate systems. Nevertheless, the epoxy/CNT core material of the microcapsules was dissolved in a solvent that is not suitable for self-healing applications below 0 °C^54^.

**Figure 1:**
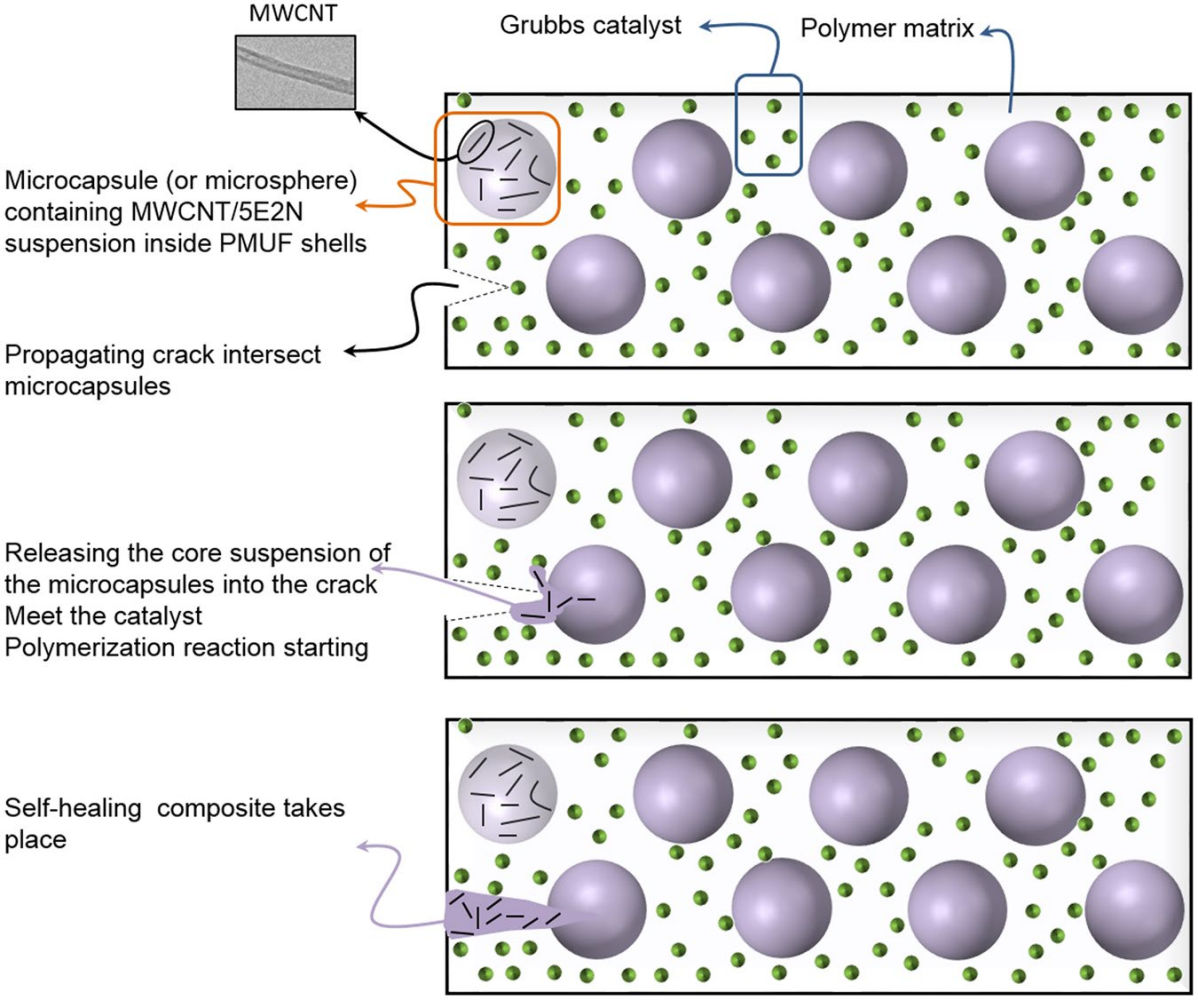
Self-healing approach based on CNT/5E2N microcapsules.

Figure 1 shows the conceptual route of the self-healing process originally introduced by White *et al*.^8^, using CNT/5E2N suspension as microencapsulated healing agents. After encapsulation of the CNT/5E2N suspension into spherical PMUF shells, the microcapsules along with Grubbs’ catalyst were uniformly dispersed inside the polymer matrix.

When cracks form inside the composite due to ageing or damage events, they propagate towards the microcapsules, so that the shell walls of these MC break and release the CNTs with the liquid monomer into the crack through capillary action^8^. Once the CNT/5E2N suspension comes into contact with the dispersed Grubbs catalyst, the latter locally triggers the ROMP reaction. The CNT/poly (5E2N) nanocomposite forms at the crack site, which partially restores the nominal functionality of the material.

## Materials and Methods

### Materials

EPON 828 (a bisphenol-A based epoxide resin) and epicure 3046 (an amidoamine room temperature curing agent) were purchased from Miller Stephenson Chemical Co.

MWCNTs were purchased from Bayer Material Science. These MWCNTs feature diameters in the 2–20 nm range, and lengths between 1–10 µm with 95% purity. The self-healing 5E2N monomer was made from a mixture of endo and exo, 99%, containing 100–200 ppm Butylated hydroxytoluene (BHT). The 2^nd^ generation Hoveyda-Grubbs (HG2) catalyst, the encapsulating shell materials melamine, formaldehyde, urea, as well as the emulsifying agent polyvinyl alcohol (PVA) and the surfactant sodium lauryl sulphate (SLS) were purchased from Millipore Sigma, Canada. All chemicals were used as received. We also used a conductive commercial silver/epoxy ECCOBOND 56 C (Henkel Loctite Ablestik 56 C)/Catalyst 9, purchased from Ellsworth Adhesives, as electrodes for electrical conductivity measurements.

### Characterization

The size and surface morphology of the microcapsules implemented in this work were investigated at various magnifications using an Optical Microscope (OM), a field emission JEOL JSM7600F electron microscope (FESEM) at an acceleration voltage of 5 kV, and a JEOL JEM-2100F transmission electron microscope (TEM), operating at 200 kV, respectively. The measurement of the microcapsules average diameter was obtained using the image analysis freeware: ImageJ.

Thermogravimetric analysis (TGA) and differential scanning calorimetry (DSC) analysis were performed to determine the thermal stability of the microcapsules. For the TGA analysis, the microcapsule samples were heated from 25 to 700 °C under helium environment, using a heating rate of 10 °C/min thanks to a TGA Q500 instrument. For the DSC analysis, the samples were heated from 0 to 100 °C (under nitrogen environment) at a heating rate of 10 °C/min using a Mettler Toledo DSC system. All the data acquired from TGA and DSC testing were analyzed using the ‘TA Universal Analysis’ software. A Leica Optical Systems Series DM LM Raman spectrometer was employed to monitor *in situ* the ROMP reaction of 5E2N initiated by the HG2 catalyst. This apparatus was equipped with a thermoelectrically cooled charge coupled device (CCD) detector and a 600 lines/mm diffraction grating to collect data over a spectral range of 300–3300 cm^−1^. A 785 nm laser line was used for optical excitation whose intensity of 0.5 mW was set to prevent sample damaging during the measurements. The Raman characterizations were performed using a confocal Invia Renishaw RM 3000 spectrometer, equipped with a digital camera and a 50× objective lens of 0.75 numerical aperture (NA) to study and compare the spectral signatures of microcapsules with and without CNTs.

### Recovery of electrical and mechanical properties

The mechanical healing efficiency of the polymer composites are determined by measuring the recovery of fracture toughness^6–9,11,12^ of the damaged samples according to the test protocol established by the research group of White *et al*.^8^. Cured epoxy samples of tapered double-cantiliver beam (TDCB) fracture geometry, incorporated into the medium with CNT/5E2N microcapsules and HG2 catalysts are prepared and tested under mode I fracture protocol^21^. The four studied samples were manufactured with 10 wt.% of microcapsules of 70 μm average diameter and 1 wt.% of HG2 catalyst. With this amount of microcapsules, the weight percent of CNT in the whole epoxy matrix would be about 0.008 wt.% (See the calculation detailed in the supplementary information section). Reference neat TDCB epoxy samples, containing no microcapsules and catalysts, as well as epoxy samples incorporated with catalyst and microcapsules containing 5E2N only were also manufactured and tested for comparison purpose. All the studied materials were first loaded until their complete failure, to be then allowed to heal for 24 hours at  RT, and reloaded for evaluating the healing efficiency.

During these experiments, the mechanical healing efficiency, ${\eta }_{h,m}$, is determined as^21^

1 $${\eta }_{h,m}=\frac{{K}_{Ic,healed}}{{K}_{Ic,virgin}}=\frac{{P}_{C,healed}}{{P}_{C,virgin}}$$

where, ${K}_{Ic,virgin}$ is the fracture toughness of the virgin specimen, ${K}_{Ic,healed}$ is the fracture toughness of the healed specimen, and ${P}_{C}$ is the corresponding critical failure loads of the virgin and healed specimens. The geometry of the samples are detailed in the supplementary information section.

The self-healing ability of microcapsules with and without CNTs to restore the electrical conductivity of silver/epoxy polymer containing HG2 catalyst were also fabricated and tested. Detailed information regarding the preparation of these samples, as well as the experimental tests and analyses that have been conducted are described in the supplementary information section.

## Results and discussion

### Fabrication of CNT/5E2N microcapsules

Microcapsules containing MWCNTs suspended in 5E2N were successfully synthesized by *in situ* polymerization following the process developed in ref. ^27^. Controlled dispersion-emulsification-polymerization-recuperation steps were carried out to enable the microencapsulation of CNT/5E2N suspension in PMUF shells. The MWCNTs were first dispersed into the 5E2N monomer using sonication, where the power and duration were the main controlling factors in obtaining a stable CNT/5E2N suspension. Figure A1 in the appendix shows that the CNT/5E2N suspension remains stable even after one week. During emulsification, shell materials (melamine, formaldehyde and urea), core material (CNT/5E2N suspension), emulsifier (PVA) and surfactant (SLS) were mixed in an aqueous medium. Both the stirring speed and the concentration of emulsifier and surfactant were found to be critical to produce oil-in-water emulsions with the steady presence of micro droplets of the core material. During the polymerization step, the emulsion was heated to the reaction temperature of 86 °C at a heating rate of 2 °C/min, according to the process presented in ref. ^27^.

The temperature was found to strongly influence the formation of urea formaldehyde (UF) micro particles, as well as the thickness, roughness and porosity of the microcapsule shells^55^. The reaction was then kept running at this temperature for about five hours. During the collection of the microcapsules, the emulsion was first cooled down, rinsed several times with deionized water and acetone, and filtered. The synthesized microcapsules were then dried in air and stored in glass vials before and between each characterization. The combination of the main fabrication parameters that resulted in the successful microencapsulation of CNT/5E2N is presented in Table 1.

**Table 1:** Controlling parameters for successful CNT/5E2N microencapsulation.

Parameters	Amounts
CNT	0.1 wt. %
Sonication power	6 W
Sonication time	2 h and 30 min
Stirring speed	700 rpm
PVA	6.3 wt. %, Molecular weight: 85000–124000
SLS	0.75 wt. %

### Microscopic investigations

**Figure 2:**
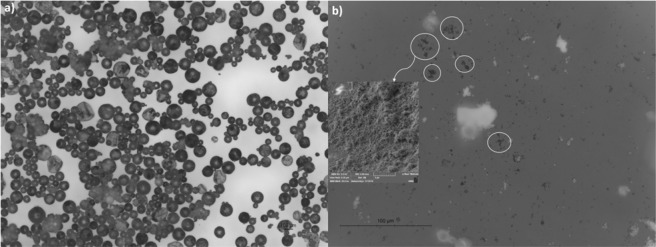
(**a**) Free flowing microcapsules observed under OM (5X). (**b**) Released CNT particles (encircled) as observed on the glass slide after crushing the microcapsules (OM 50X). The inset shows the high magnification (25000X) image of CNTs released.

Figure 2a shows a typical optical microscopy image of the individually separated spherical free-flowing microcapsules. In Fig. 2b, the white arrows point CNTs, which have been released within the liquid core upon the crushing of few milligrams of microcapsules. Reference microcapsules that contain only 5E2N (i.e. without CNTs) do not show such features. Further, the liquid core content of the microcapsules is isolated and the released CNTs are seen under SEM, as shown in the inset of Fig. 2b.

**Figure 3:**
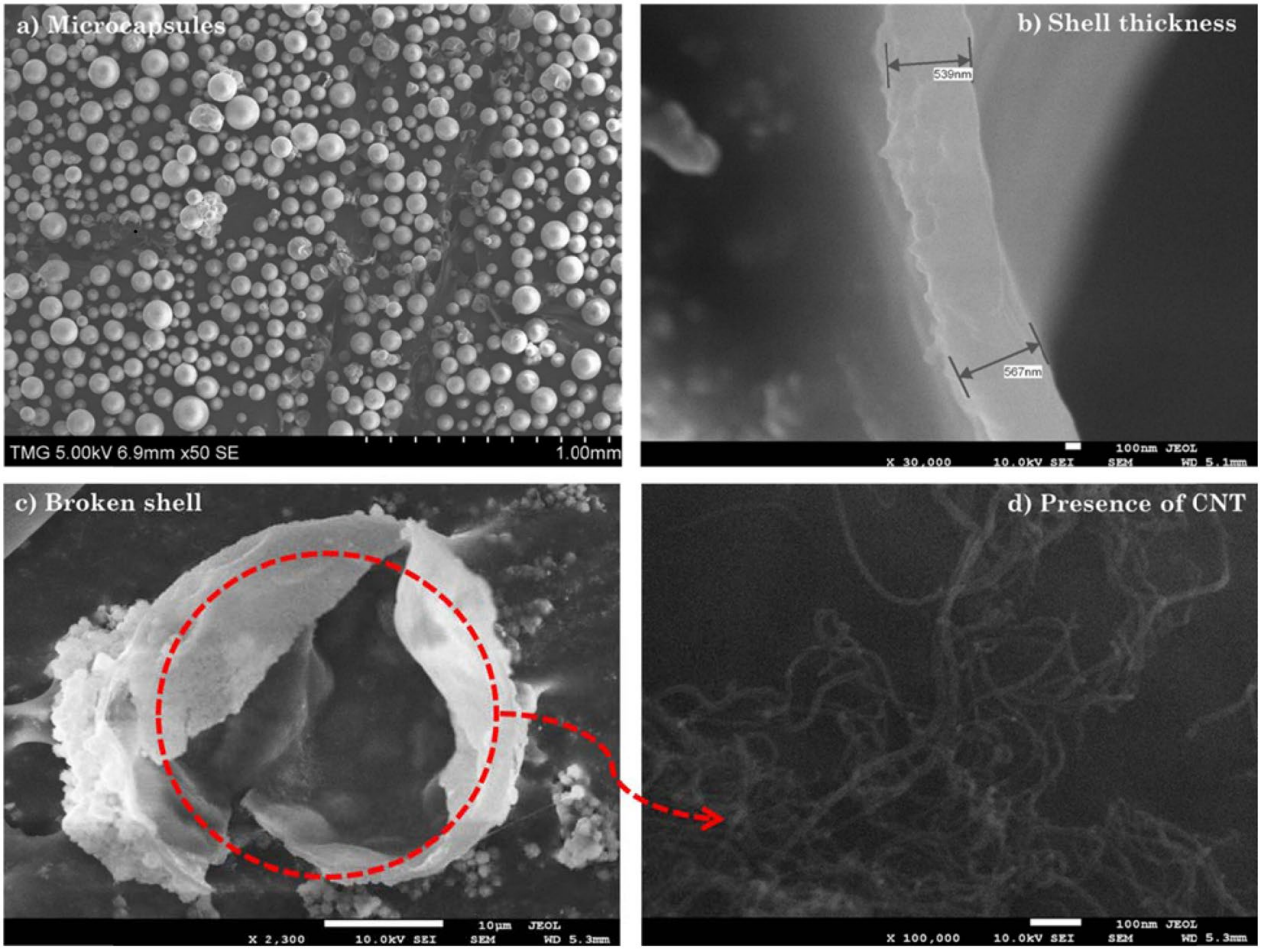
SEM observations of (**a**) individually separated spherical microcapsules (**b**) shell thickness (**c**) core-shell structure (**d**) CNTs inside the core of the microcapsules.

Figure 3(a–d) shows representative SEM micrographs obtained at various magnifications: x50, x30000, x2300 and x100000, respectively. Individually separated spherical microcapsules are shown in Fig. 3a, with core-shell structure displayed in Fig. 3b,c. The average shell thickness is found to be around 550 nm. This shell thickness of the microcapsules is sufficiently robust to survive handling and manufacturing of self-healing polymer composites^17^. The outer shells of the microcapsule walls are observed to be textured, as shown in Fig. 3c, with nanoparticles deposited onto its surface during the *in situ* polymerization process^17^. The white structures observed in Fig. 3d and pointed by arrows are CNTs, whose metallic properties make them brighter than the background contrast under electron beam exposure^56^.

**Figure 4:**
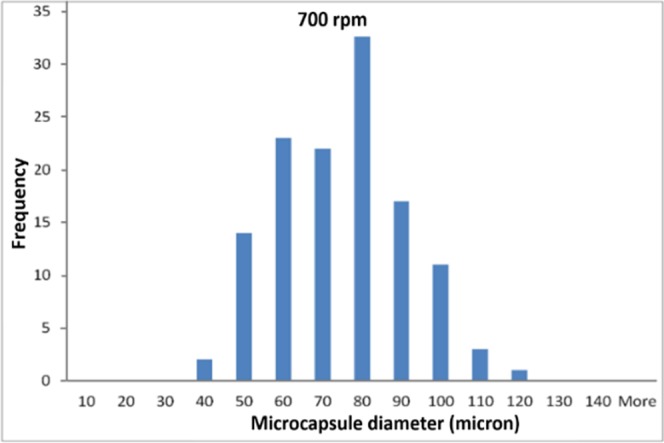
Typical size-distribution histogram of CNT/5E2N microcapsules.

Figure 4 displays a typical size-distribution histogram of the microcapsules produced at 700 rpm stirring speed. Their diameters vary between 40 and 120 µm, around an average value of 70 µm. Both the dimensions and the size-distribution of the microcapsules strongly depend on the stirring speed^17^ that was selected during the synthesis process.

The uneven sizes of the microcapsules may affect the reproducibilty of the self-healing efficiency especially at the nanoscale, because the core material could be not uniformly released. To minimize this effect, microcapsules of desired size-range can be sort using micro-sieves and their concentration in the composite can be increased.

**Figure 5:**
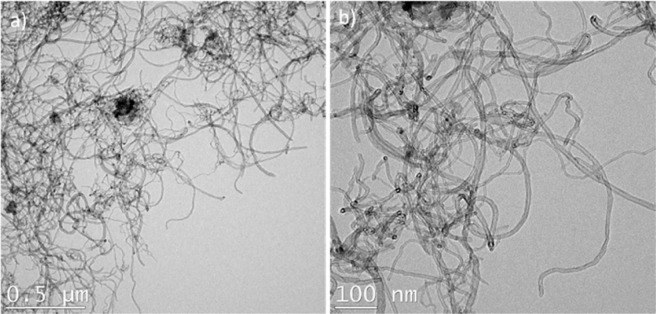
TEM observation of CNTs in the core liquid of the microcapsules.

For TEM observations, we extracted the liquid released from few milligrams of crushed microcapsules and dissolved it into acetone and methanol. A tiny Cu grid with carbon film was then immersed into the solution and dried before placing it into the TEM. The TEM micrographs presented in Fig. 5 show the presence of CNTs in the liquid core extracted from the crushed microcapsules. These images thus confirm that the processing steps of the synthesis of microcapsules did not cause severe breakage to the CNTs and maintain their original high aspect ratios. Such features are very important for maximizing the benefits of incorporating CNTs/5E2N into the core of the microcapsules, especially in terms of their electrical properties.

### Thermal stability analysis

**Figure 6:**
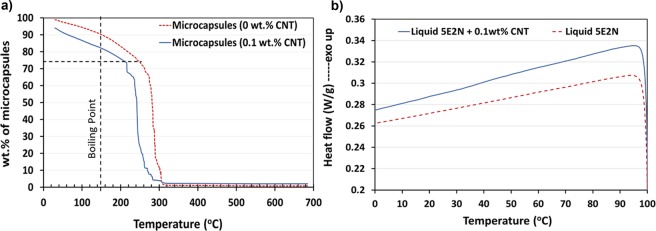
(**a**) Comparison of weight loss vs. temperature in TGA for microcapsules containing liquid 5E2N only, and microcapsules containing 5E2N/CNT suspension with 0.1 wt. % CNT. (**b**) Comparison of heat flow vs. temperature of liquid 5E2N and CNT/5E2N suspension with 0.1 wt. % CNT.

TGA was carried out separately for reference microcapsules containing only 5E2N and microcapsules containing CNT/5E2N suspension. During these thermogravimetric experiments, the weight-loss of the samples was measured while the temperature was increased, as reported on Fig. 6a.

The drawn dashed vertical line reported on Fig. 6a is connected to the boiling point of 5E2N (148 °C)^57^, whereas the dotted horizontal line refers to the points on the curves where rapid weight loss begins. According to Liu *et al*.^27^, the abrupt drop of the weight loss, observed around 240 °C for reference samples, can be associated with the sudden burst of the microcapsule shell. This rupture is caused by the buildup of the vapor pressure inside the microcapsules. The tearing of the microcapsule shells releases the vaporized core materials in the TGA cell, thus resulting in rapid weight loss. For CNT/5E2N microcapsules, this phenomenon also occurs at lower temperatures, around ~220 °C.

The dispersion of CNTs within the liquid 5E2N was found to affect the thermal stability of the medium. This effect is confirmed by the DSC analysis presented in Fig. 6b, where drops of liquid 5E2N and 0.1 wt. % CNT/5E2N suspension were heated separately from 0 to 100 °C. The difference in the slopes of the two lines (Fig. 6b) indicates the difference in the thermal properties for the 5E2N/CNTs, which possibly causes a quicker build-up of the vapor pressure inside the microcapsules. The presence of 0.1 wt. % CNTs inside the 5E2N liquid slightly decreases the maximum temperature to which the microcapsules can be heated before breaking. Moreover, even if this limit is somehow lower than that of the microcapsules containing only 5E2N, the thermal stability of about 220 °C makes the CNT/5E2N microcapsules still suitable for use in epoxy polymer composites, which may require curing at elevated temperatures (up to 180 °C) for some applications^37^. On the other hand, the operation temperatures in satellites and other space vehicles do not usually exceed 125 °C^58–60^. This suggests that the 5E2N/CNT microcapsules fabricated in our work are stable and operational in the thermal environment of an orbiting spacecraft.

### Raman analysis

**Figure 7:**
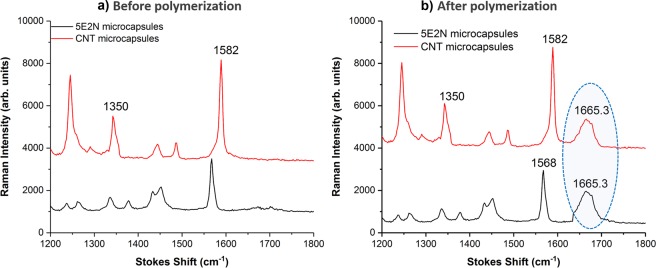
Comparison of Raman spectra generated for 5E2N microcapsules and 5E2N/CNT microcapsules, before (**a**) and after (**b**) polymerization.

Figure 7 shows the Raman spectrum of the core liquid extracted from the reference microcapsules and the CNT/5E2N microcapsules, before (Fig. 7a) and after the polymerization process (Fig. 7b). The Raman peak observed around 1350 cm^−1^ (Fig. 7) corresponds to the spectral signature of the disorder-induced mode ‘D-band’ of CNTs. This mode is related to the presence of disorder in sp^2^-hybridized carbon systems, whose out-of-plane vibration is more prominent for MWCNTs^61^. The phonon peak at 1582 cm^−1^ refers to the ‘G-band’, which is connected to tangential in-plane vibrations of sp^2^-bonded carbon atoms^61–66^.

These two representative Raman signatures confirm the presence of MWCNTs inside the CNT/5E2N microcapsules. None of the D band and G band peaks have been observed in the reference microcapsules, where the Raman phonon at 1568 cm^−1^ is attributed to the C=C stretching vibration of the norbornene ring of 5E2N^65,67^. Due to the addition of the catalyst to the core liquid of the microcapsules, another Raman peak appears around 1665.3 cm^−1^ (as indicated within the ellipse in Fig. 7b). As previously reported, this peak confirms the formation of poly (5E2N) after ROMP polymerization reaction for both types of microcapsules^10^.

### Recovery of fracture toughness

**Figure 8:**
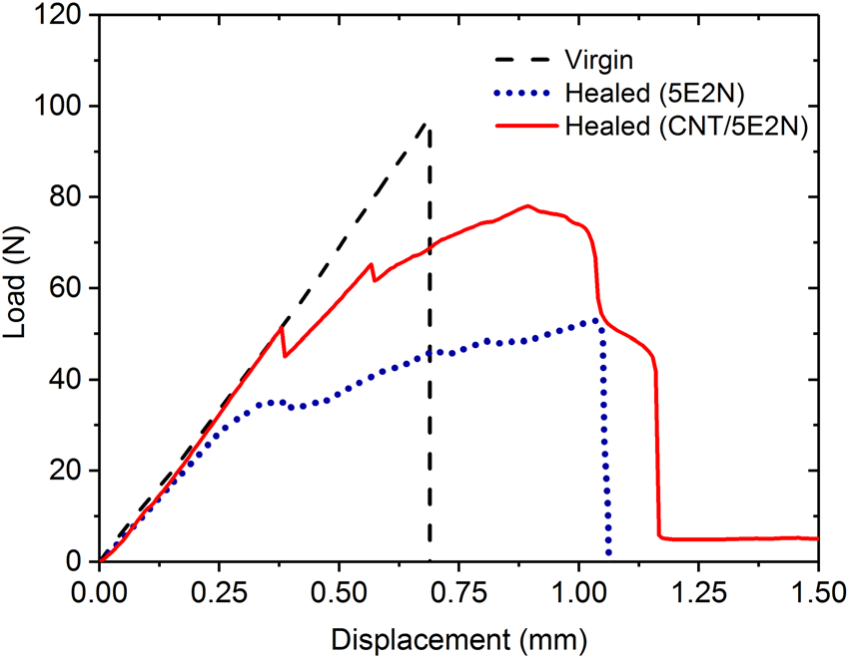
Representative load-displacement curves for TDCB samples in mode I fracture test.

The representative load-displacement curves of TDCB epoxy samples where pure 5E2N and CNT/5E2N microcapsules have been incorporated inside the medium with HG2 catalysts are shown in Fig. 8 for mode I fracture tests.

Our measurements show that during the first loading, often named as ‘virgin loading’ in the literature^16–22^, the TDCB sample undertakes increasing load as the displacement is increased. As the load approaches to the peak load (around ~100 N in Fig. 8), the mode I crack is generated in the sample and propagates along the centreline (Fig. A2 in supplementary section) through the entire length of the sample leading to its complete failure. At this stage, the TDCB sample is separated into two parts and its load carrying capacity drops to zero. The two halves of the damaged TDCB sample are then put into contact for 24 hours during which the self-healing reaction occurs. After this period, the two halves of the TDCB sample are observed to be glued together and the reassembled sample is loaded for a second time (labelled as ‘healed’ in Fig. 8) similarly to the first loading, in order to determine its load carrying capacity after healing. It is to be noted that, the reference neat TDCB epoxy samples that do not contain any microcapsules and catalysts, could not carry any further load after their complete failure during the first loading. As expected for these materials, the two halves of the TDCB reference samples remained separated after 24 h and could not even be reloaded as no self-healing occurred.

The comparison between the load-displacement curves of the virgin and healed samples reveals that the healed samples containing CNT/5E2N microcapsules are tougher, so that they can withstand more displacement before their final failures. This feature is consistent with highly crosslinked epoxy, which is naturally rigid and brittle^68^. When a crack is once again initiated inside the healed samples, its propagation is less uniform than within virgin epoxy samples due to the presence of healed CNT/poly (5E2N) nanocomposites inside the medium. Consequently, the load-displacement curves of the healed CNT/5E2N samples shown in Fig. 8 do not increase continuously with the displacements. This also favours  the occurrence of a stick-slip fracture behavior^69^, characterized by a repetitive unstable propagation of the crack followed by a period of crack stoppage through the healed materials. These Stick-slip fracture behaviours in adhesives under mode I fracture conditions have already been observed in ref. ^70^, where the substrate roughness is believed to play an important role at the microscopic level. In the adhesive layer, the bondline discontinuities^71^, even defects^72^ result in the fluctuation of fracture energy that can lead to stick-slip fractures. According to ref. ^73^, the non-linear behaviour of the load-displacement curve at the end of the rising stage originates from the crack formation process and changes in the shape of the crack front. At the microscale, these cracks were found to propagate in a zigzag way through the self-healed adhesive^74^, which can be regarded as low-grade crack deviations that also helps increase the fracture loads.

**Figure 9:**
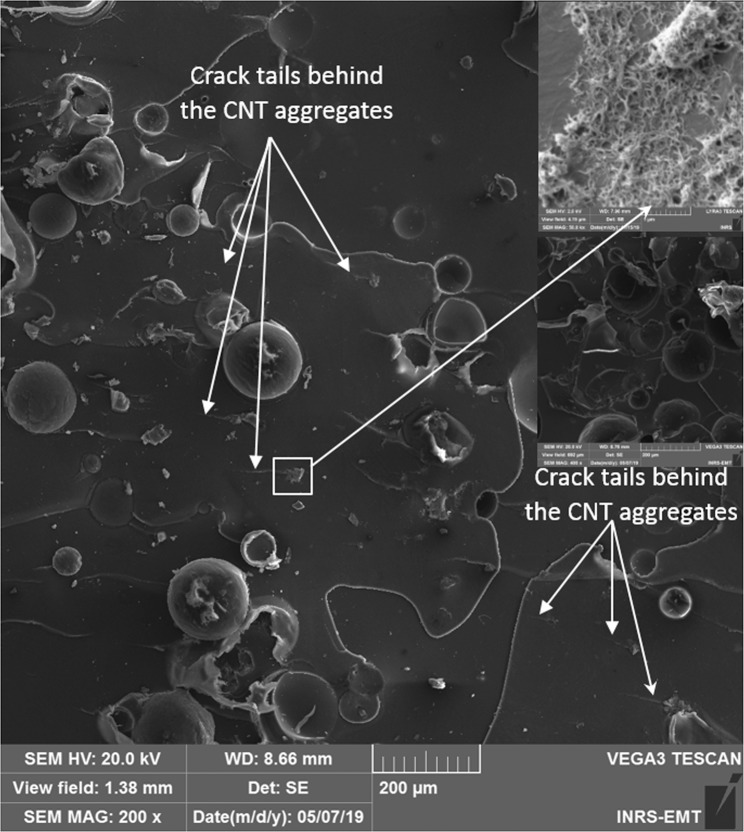
Representative SEM micrograph of fracture surface of TDCB samples after the mode I fracture test. CNT aggregates at the fracture surface are shown in the inset. Presence of crack-tails indicate crack pinning mechanism of toughening by the CNT aggregates.

Last but not least, the inclusion of CNT fillers into the cracks may also affect its propagation^75^. To further verify this effect, the fracture surface of the TDCB samples has been examined by SEM. A representative SEM micrograph of the fracture surface of the TDCB samples is presented in Fig. 9, showing distinct poly (5E2N) film and broken microcapsules that have released their core content CNTs (shown in the inset).

In addition to confirming that the self-healing ROMP reaction is activated at the fracture surface where poly (5E2N) films are produced to glue and heal the crack, this figure also shows the presence of tails behind the CNT aggregates, as pointed by arrows at many different locations inside the poly (5E2N) layer. These features of crack are attributed to CNT aggregates, whose effect on the composite toughening can be associated with a crack pinning mechanism^20,68^. Such a phenomenon is caused by the bifurcation of the crack propagation path in the presence of the filler surface, followed by the subsequent meeting of the previously separated crack fronts^68^.

The dispersion of CNTs in monomer followed by *in situ* polymerization favours the formation of CNT/Polymer nanocomposites. This results in a stronger and more active interface between the CNTs and polymer, which is the key for improving the structural performance of these composites^76^. According to ref. ^25^, the incorporation of CNT fillers into 5E2N monomer may act as a cross-linking network in poly (5E2N)^25^, leading to a significant increase of its mechanical properties such as hardness, strength and stiffness.

Although the wt. % of CNTs in the whole epoxy matrix is only about 0.008 wt. %, the microcapsules enable the release of a well-dispersed suspension of CNT/5E2N into the crack sites. This ensures that a sufficient amount of CNTs come into contact with 5E2N. Their subsequent in situ polymerization produces strong poly (5E2N)/nanotube interfaces as evidenced in Fig. 9. If CNTs are pre-dispersed into the epoxy matrix and 5E2N-only microcapsules are used instead, they  might not be liberated at the crack site and they are less likely to form an effective dispersion with the released 5E2N resulting in weaker poly(5E2N)/nanotube interfaces.

**Figure 10:**
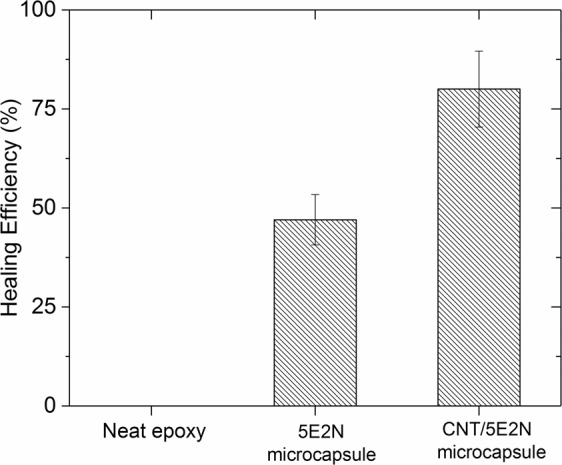
Comparison of mechanical healing efficiency of three types of TDCB epoxy samples, reference (neat epoxy) samples containing no microcapsules, samples containing 5E2N microcapsules and samples containing CNT/5E2N microcapsules.

The average healing efficiencies achieved by the three types of tested samples, as calculated from Eq. (1), are compared in Fig. 10, showing that, in absence of any microcapsule and catalyst, the reference neat epoxy samples could not recover any fracture toughness. On the other hand, the samples containing catalyst and 5E2N-only microcapsules recover about 47% of their nominal fracture toughness, and the samples containing catalyst and CNT/5E2N microcapsules, about 80%. Such results prove indeniably that the presence of CNTs inside 5E2N significanlty improves the restoration efficiency of the structural integrity of self-healing composites, in agreement with the data  reported in ref. ^25^.

Rule *et al*.^16^, who studied the effects of size and wt.% of microcapsules containing DCPD on the healing performance of epoxy showed that the healing efficiency can be optimized by choosing different combinations of microcapsule size and concentration for given sizes of cracks to be healed. According to the authors^16^, a high concentration (e.g. 20 wt%) of large (up to 400 μm) microcapsules can deliver more healing agents into the cracks for better healing. However, high concentration of larger microcapsules might negatively affect the virgin properties of the epoxy^20^, as well as raise some manufacturing issues related to the increase of the resin viscosity, and  difficulties in achieving  uniform dispersion. From our results, we recommend to increase the concentration of CNTs in the microcapsule core to release more CNTs into the crack and produce more poly (5E2N)/CNT interfaces, so that the healed material will be toughened  through the crack-pinning mechanism occuring in more sites, as shown in Fig. 9

### Recovery of electrical conductivity

The electrical self-healing ability of CNT/5E2N microcapsules is  evaluated using the samples fabricated with electrically conductive epoxy adhesives (Eccobond 56 C) and incorporated separately with two types of microcapsules. Neat conductive samples without microcapsules were also fabricated as reference. Wheatstone bridge circuits, whose basic principle and operation are presented in the ESI section (see Figs. A3 and A4) are installed on the samples. The normalized bridge voltages (see Eq. (3) in ESI) are measured and compared for evaluating the electrical healing efficiency of the samples. Details regarding  the fabrication of samples and  the test procedures are given in the ESI section.

**Figure 11:**
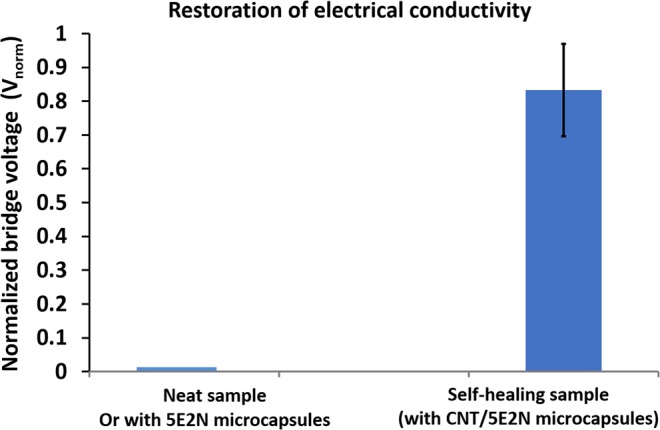
Comparison of the restoration of electrical conductivity of the neat (no microcapsules) and self-healing (with microcapsules) samples.

The Fig. 11 demonstrates two types of electrically conductive samples whose conductive paths were first disrupted by introducing a damage/cut (cutting with razor blade) to the conductive path so that no electrical current could pass through it. The samples were then left untouched for 24 hours to allow for self-healing. The samples which did not contain any microcapsules or contain 5E2N-only microcapsules, could not recover the conductivity. The samples that contain the CNT/5E2N microcapsules are found to be able to recover their electrical conductivity, up to 82% of its nominal value as shown in Fig. 11. Despite possible variations due to the presence of microcapsules non-uniform in size, no significant change in electrical properties is recorded in the studied samples after healing. This may result from the very low percolation threshold for electrical conductivity inside the CNT/polymer composites^76^.

This makes the incorporation of CNTs into 5E2N monomer relevant to restore the electrical conductivity and/or connections inside damaged electronic circuits. The microcapsules containing such CNTs can thus permit a better repairing/restoring of key electronic components and/or optoelectronic devices. Aïssa *et al*.^77^ demonstrated that the performance of a flexible fluidic antenna incorporated with conductive nanocomposite materials are greatly influenced by the electrical conductivity of the nanocomposites.

In addition, the mechanical properties of the nanocomposite materials are found to contribute significantly to the overall elasticity of the antenna structure, which can be deformed and/or bent repeatedly without losing its performance. The CNT/5E2N microcapsules offer the possibility of restoring significantly both the electrical conductivity and the structural integrity of systems that may degrade due to aging effects and damaging events occurring in harsh space environments. Since the 5E2N healing agent remains active below 0 °C^10^ and the presence of CNTs inside encapsulated 5E2N does not usually affect the kinetics of the ROMP reaction, as reported in the Fig. A5 of the ESI section for CNT/5E2N with Grubbs catalyst, we infer that the improvement of self-healing abilities due to the presence of CNTs is relevant for applications in space environment. This provides an alternative solution to extend both their lifetime and their reliability, even for operation at low temperatures and/or temperature gradients similar to space environment conditions.

## Conclusions and Perspectives

MWCNTs suspended in 5E2N self-healing monomer capable of undergoing ROMP reaction were successfully encapsulated using an *in situ* polymerization technique. An experimental trial and error procedure was used to identify suitable process parameters including the wt. % of CNT, both the power and duration of sonication for dispersion, as well as the concentration of emulsifying agent and surfactant. After successful encapsulation of 5E2N/CNT suspensions, the average dimension, size-distribution, shell-wall topology and thickness of the microcapsules were characterized by microscopic observations. Optical, SEM and TEM images show the formation of microcapsules, as well as the presence of CNTs inside their core and the release of their content upon crushing. As confirmed by Raman analysis, the ROMP reaction is observed for pure 5E2N and mixed CNT/5E2N microcapsules. The structural integrity of these CNT/5E2N microcapsules was investigated up to 600 °C, showing that such system can resist to external temperatures of 220–250 °C, which makes them suitable to be used in advanced polymer composites for aerospace. After testing, 80% of the fracture toughness and 82% of the electrical conductivity are found to be autonomically restored inside the polymer composite containing 5E2N/CNT self-healing microcapsules. These new 5E2N/CNT PMUF shells offer unique self-healing solutions to devices/structures that require restoration of either or both electrical and mechanical properties with efficient functioning, even at low temperatures. Their ease of being integrated into a great number of composite materials makes them suitable to protect and extend the lifetime of telecommunication equipment and sensors exposed to accidental events and collisions with small debris, as well as critical ageing effects that can cause their irreversible damage and failure. The use of encapsulated 5E2N/CNT could provide a significant added-value to commercial products and devices requiring long operation periods inside facilities or environments where they cannot be repair and/or replaced by new ones. Example of such devices/structures are flexible fluidic antennas, where both electrical conductivity and mechanical properties ensure their efficient functioning, to be used in wireless sensing or monitoring radio systems, switches, radio frequency identification (RFID) tags, conformal circuits for health monitoring or in other military and space applications.

## Supplementary information

Supplementary information.
