# Supplementary material for: Recovery of electro-mechanical properties inside self-healing composites through microencapsulation of carbon nanotubes
Source: Sci Rep. 2020 Feb 19;10:2973. doi: 10.1038/s41598-020-59725-6 (PMC7031524; doi:10.1038/s41598-020-59725-6)
Supplement: Supplementary file 1 — Supplementary information. [file 41598_2020_59725_MOESM1_ESM.pdf]

Supplementary Information

Recovery of electro-mechanical properties inside self-healing composites through microencapsulation of carbon nanotubes

Hasna Hena Zamal [1], David Barba [1], Brahim Aïssa [2, 3]*, Emile Haddad [2], Federico Rosei [1]*

[1] Institut National de la Recherche Scientifique, Centre: Energie, Matériaux et Télécommunications, 1650 Boulevard Lionel-Boulet, Varennes (Québec), J3X 1S2, Canada, E-mail : rosei@emt.inrs.ca, Tel.: +1-514-2286906; Fax. +1-450-9298102

[2] MPB Communications Inc., Space & Photonics Division, 151 Hymus Boulevard, Pointe Claire, Montréal QC, H9R 1E9, Canada

[3]Qatar Environment and Energy Research Institute (QEERI), Hamad Bin Khalifa University, Qatar Foundation, 5825, Doha, State of Qatar
E-mail: brahim.aissa@mpbc.ca, Tel.: +1-438-4489085; Fax. +1-514-6946869

Stability of CNT/5E2N suspension

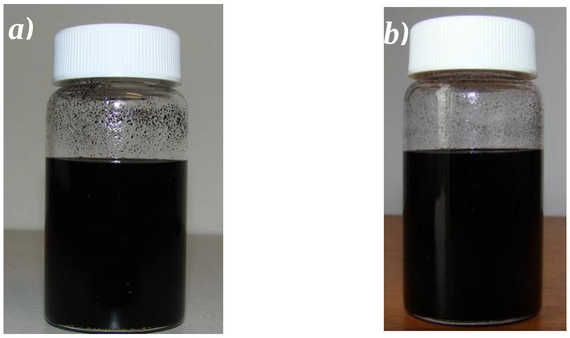

Figure A1. CNT/5E2N suspension a) right after the sonication and b) after 7 days

TDCB samples preparation:

Figure A2 shows the geometry of tapered double-cantilever beam (TDCB) epoxy specimen. The test is performed by the MTS machine with a load cell of 1KN, at a crosshead speed of 0.5mm/min. Four samples were manufactured with 10 wt. % of microcapsules of average size 70 μm and 1 wt. % of HG2 catalyst in epoxy and curing agents mixed at a concentration of 100:47. The mixtures are cured at room temperature for 10 days for complete curing according to the

recommendation of the curing agent supplier. After 10 days, the samples are post cured at elevated temperature (100 ^oC) for 4 hours.

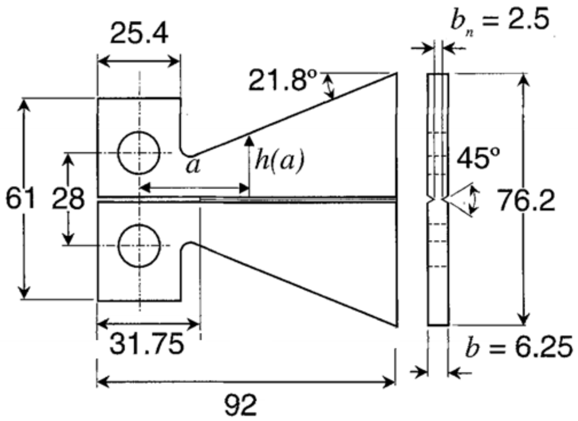

Figure A2. Tapered double-cantilever beam (TDCB) geometry (dimensions in mm) [1]

wt. % of CNT in the whole epoxy matrix

wt.% of microcapsules dispersed into epoxy, w_{mc} = 10 wt. %

The core content (CNT/5E2N suspension) of the microcapsules, w_{core} = 80-90% (approximately) of the weight of the microcapsules [From TGA analysis shown in figure 6.a in the MS].

wt.% of CNT in the core suspension, $w_{CNT,\ core}$ = 0.1 wt.%

wt. % CNT in the whole matrix, $w_{matrix} = w_{mc}$ X w_{core} X $w_{CNT,\ core}$

= 0.008 wt% (approximately).

Fabrication of self-healing composite samples for electrical conductivity restoration tests:

To investigate the ability of microcapsules to restore electrical conductivity in damaged materials, specific samples are prepared with conductive silver/epoxy polymer and self-healing composite microcapsules. Neat samples (containing no microcapsules) are obtained by mixing ECCOBOND 56C and catalyst 9 in a weight ratio of 100:2.5. The mixture is then spread on a polymer substrate to make a conductive path and cured for 24 hours at room temperature followed by one hour of thermal annealing at 60 ^oC. Two electrical connections are attached to

two different points along the conductive path [as shown in Figures A3 (a) and (c)] of the samples. The samples are connected later to a Wheatstone bridge circuit (which is a widely used technique for accurate measurements of electrical signals even for a slight change in resistance) for voltage measurements. The self-healing samples (with microcapsules) are prepared similarly by mixing and curing a pre-specified amount of ECCOBOND 56C, catalyst 9, CNT/5E2N microcapsules and Grubbs catalyst. After curing, the conductive path of the samples is cut with a razor blade [figures A3 (b) and (d)]. The samples are then left for 24 hours at room temperature for healing. The bridge voltage is measured i) at the beginning (uncut sample), ii) after cutting the conductive path, and iii) after allowing 24 hours to heal (for the self-healing sample) the cut.

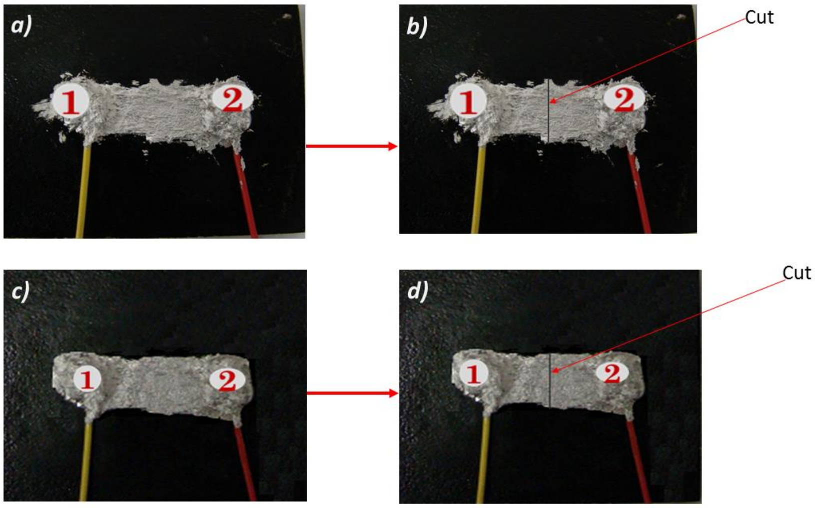

Figure A3. Procedure to determine the self-healing ability of CNT/5E2N microcapsules to restore electrical conductivity of composite samples. Bridge voltages are measured for the neat (no microcapsules) and self-healing samples (with microcapsules) with a) and c) uncut conductive path; and b) and d) cut between point 1 and 2 and after allowing for healing for 24 hours.

Measurement principle and determination of self-healing efficiency:

The restoration of electrical conductivity of the silver/epoxy samples is evaluated in accordance with the procedure described in Ref. [3]. The Wheatstone bridge circuit consists of three standard resistors: 100 Ohms (R_1), 10 Ohms (R_2), and 250 Ohms (R_4) resistance, with an input voltage (V_{in}) of 5.0 V. The silver epoxy sample is connected to the circuit as another resistor (R_3), as shown in figure A6. For a slight change in the electrical resistance of the samples connected to

the Wheatstone bridge circuit due to the disruption/restoration of the conductive path, the output bridge voltage (V_g) changes accordingly.

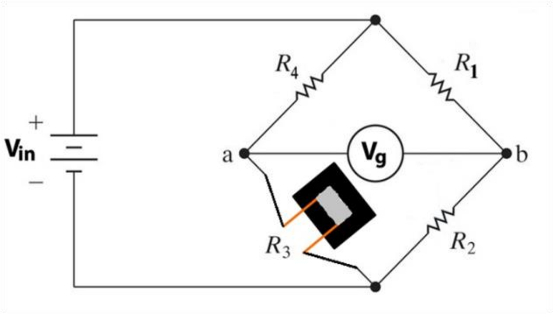

The bridge voltage (**V_g**) for all condition is measured via connection between **a-b**

Figure A4. Circuit diagram of the Wheatstone bridge, the sample is connected in the position of the R3 resistor [3].

Three bridge voltages (V_g, V_c and V_h) are measured. V_g is the initial bridge voltage, V_c is the bridge voltage when the samples are cut and V_h is the bridge voltage after the samples are allowed to heal for 24 hours.

From the circuit analysis, the bridge voltage V_g is given in terms of the resistances [1] by:

$$V_g = V_{in} [(R_1/ (R_1 + R_2)) - (R_4/ (R_3 + R_4))] \text{ ………… Eq. (1)}$$

The bridge voltage for the cut samples V_c,

$$V_c = (V_{in} \text{ x } R_1)/ (R_1 + R_2) \text{ ....………………………… Eq. (2)}$$

Autonomic restoration of electrical conductivity (healing efficiency) is assessed in terms of

V_{norm},

$$V_{norm} = (V_h - V_c)/ (V_g - V_c) \text{ ………………………… Eq. (3)}$$

$$= (R_3 + R_4)/ (R_h + R_4),$$

where R_h is the resistance of the samples after healing.

In damaged samples, no electrical current pass and the cut bridge voltage is given by Eq. (2). For the healed samples, when the current can flow through the circuit, the voltage is dropped and

given by Eq. (1). Here, the normalized bridge voltage of the samples as calculated by Eq. (3) is taken as the percentage of restoration of electrical conductivity, which defines the healing efficiency.

ROMP of 5E2N as a function of reaction temperature and CNT loads:

To point out the kinetics of the polymerization reaction as a function of the temperature and the CNT concentrations, we have performed ROMP reaction tests at temperatures ranging from -15 to 45 °C, and CNT loads ranging from 0 to 5 wt.%. A Tenney Junior Environment Chamber™ was used for this test. Figure A5 illustrates the time needed for the 5E2N polymerization (ROMP) as a function of the reaction temperature. This time is found to be 146 min at -15 °C, while it decreases to 4 min only when the reaction temperature increases to 20 °C. The polymerization occurs then in very short time (as low as 0.2 min) when the temperature is increased to 45 °C. It is worth noting here that no significant change in the kinetics of the ROMP reaction was observed with respect to the CNT loads (time values are within the experimental uncertainty error bars). In sum, at room temperature, the ROMP reaction of the 5E2N monomer triggered by the Grubbs catalyst is found to happen at very short time scales (less than 5 min). The 5E2N monomer provided definitely the best polymerization time over the alternative monomers [4].

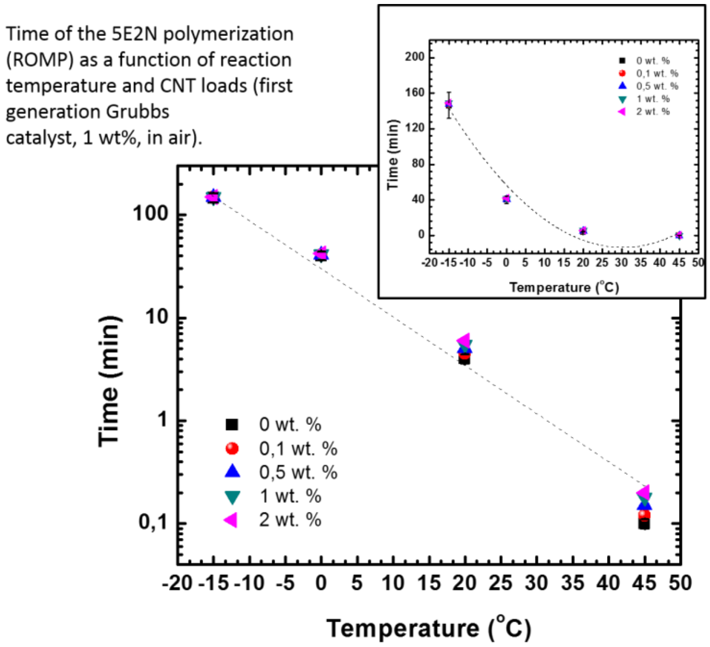

Figure A5. ROMP reaction of 5E2N as a function of reaction temperature and CNT loads.

References (Supplementary Information):

[1] Brown, E. N., Sottos, N. R. & White, S. R. Fracture testing of a self-healing polymer composite. *Exp. Mech.* **42,** 372-9 (2002).

[2] Blaiszik, B. J. *et al.* Autonomic restoration of electrical conductivity. *Adv. Mater.* **24,** 398-401 (2012).

[3] Odom, S. A. *et al.* Autonomic restoration of electrical conductivity using polymer-stabilized carbon nanotube and graphene microcapsules. *Appl. Phys. Lett.* **101,** 043106 (2012).

[4] Wool, R. P. Self-healing materials: a review. *Soft Matter*. **4,** 400-18 (2008).
